# Typhoid Fevers

**Published:** 1889-11-16

**Authors:** 


### TYPHOID FEVERS.

Dr. Buchman, Fort Wayne, Ind., contributes a paper on "colon flushing" in typhoid fever to the New York *Medical Record*, Sept. 28, 1889. Having a case which presented all the symptoms of enteric, Dr. Buchman "concluded to wash out the colon with a large enough quantity of water to thoroughly cleanse it," and to this end procured a fountain syringe of two-quart capacity. Filling the reservoir with water directly from the hydrant, he proceeded to fill the colon. This was repeated every time the temperature reached 103deg. But a few days afterwards there were alarming symptoms indicating hæmorrhage. Nevertheless the colon flushing was continued, and the patient recovered. Dr. Buchman has also treated five other cases successfully. The conclusions Dr. Buchman arrive at are that three quarts of cold water may be passed into the colon, which will rapidly lower the temperature; that some of the water passes the ileo-cœcal valve; that tympanitic distension disappears with the passing away of the water; that putrefactive fermentation is prevented; that toxic substances are most rapidly absorbed by the cœcum; and that by washing with antiseptic waters, absorption of toxic substances are prevented. Under the circumstances stated by the author, we think the plan demands an extended trial.

